# Altered Effective Connectivity Network of the Basal Ganglia in Low-Grade Hepatic Encephalopathy: A Resting-State fMRI Study with Granger Causality Analysis

**DOI:** 10.1371/journal.pone.0053677

**Published:** 2013-01-11

**Authors:** Rongfeng Qi, Long Jiang Zhang, Jianhui Zhong, Zhiqiang Zhang, Ling Ni, Qing Jiao, Wei Liao, Gang Zheng, Guangming Lu

**Affiliations:** 1 Department of Medical Imaging, Jinling Hospital, Clinical School of Medical College, Nanjing University, Nanjing, Jiangsu Province, China; 2 Department of Biomedical Engineering, Zhejiang University, Hangzhou, Zhejiang Province, China; 3 Center for Cognition and Brain Disorders and the Affiliated Hospital, Hangzhou Normal University, Hangzhou, Zhejiang Province, China; Beijing Normal University, China

## Abstract

**Background:**

The basal ganglia often show abnormal metabolism and intracranial hemodynamics in cirrhotic patients with hepatic encephalopathy (HE). Little is known about how the basal ganglia affect other brain system and is affected by other brain regions in HE. The purpose of this study was to investigate whether the effective connectivity network associated with the basal ganglia is disturbed in HE patients by using resting-state functional magnetic resonance imaging (rs-fMRI).

**Methodology/Principal Findings:**

Thirty five low-grade HE patients and thirty five age- and gender- matched healthy controls participated in the rs-fMRI scans. The effective connectivity networks associated with the globus pallidus, the primarily affected region within basal ganglia in HE, were characterized by using the Granger causality analysis and compared between HE patients and healthy controls. Pearson correlation analysis was performed between the abnormal effective connectivity and venous blood ammonia levels and neuropsychological performances of all HE patients. Compared with the healthy controls, patients with low-grade HE demonstrated mutually decreased influence between the globus pallidus and the anterior cingulate cortex (ACC), cuneus, bi-directionally increased influence between the globus pallidus and the precuneus, and either decreased or increased influence from and to the globus pallidus in many other frontal, temporal, parietal gyri, and cerebellum. Pearson correlation analyses revealed that the blood ammonia levels in HE patients negatively correlated with effective connectivity from the globus pallidus to ACC, and positively correlated with that from the globus pallidus to precuneus; and the number connectivity test scores in patients negatively correlated with the effective connectivity from the globus pallidus to ACC, and from superior frontal gyrus to globus pallidus.

**Conclusions/Significance:**

Low-grade HE patients had disrupted effective connectivity network of basal ganglia. Our findings may help to understand the neurophysiological mechanisms underlying the HE.

## Introduction

Hepatic encephalopathy (HE) is a common neuropsychiatric complication which caused disturbance of central nervous system function in patients with acute and chronic liver disease [1]. It encompasses a broad spectrum of neurological symptom of varying severity and is classified from low-grade to high-grade HE. Even the low-grade HE is associated with poor quality of life and increased work disability [2], [3], [4], both improve after liver transplantation or reasonable medical treatment with lactulose [5] and rifaximin [6]. Therefore, it is important to diagnose and treat HE before major neurological destroy occurs. Although the exact pathophysiological mechanisms of HE remain unclear, investigators have extensively investigated this disease with the aim of developing effective therapies and monitoring the effectiveness of treatment.

Accumulating evidences from neuroimaging studies suggest that an alteration of the cortico-striato-thalamic pathway might play an important role in the HE [7], [8]. Within this model, the common radiological findings of HE are hyperintensity in the basic ganglia (especially the globus pallidus) in conventional T_1_-weighted MR images [9], and redistribution of cerebral blood flow and metabolic rate of glucose and ammonia from various cortical regions (e.g., the frontal and parietal cortices) to subcortical grey matter regions (the basal ganglia and thalamus) in position emission tomography (PET) and single photon emission tomography (SPET) [8], [10].

Resting-state functional magnetic resonance imaging (rs-fMRI) which measures spontaneous low-frequency blood oxygenation level-dependent (BOLD) fluctuations [11] may help delineate the human neural functional architecture, and has been widely used to investigate the pathophysiology of many brain diseases, such as Alzheimer's disease [12] and attention deficit hyperactivity disorder [13]. In a very recent rs-fMRI study, Zhang et al. [14] reported a widespread disrupted functional connectivity between the basal ganglia and many other brain regions in minimal HE patients. Basal ganglia are involved in many neuronal pathways related to psychomotor behavior, emotional and cognitive functions [15], and is considered to play an important role in the pathophysiology of HE [16]. Even though the basal ganglia showed disrupted functional connectivity with many other brain regions [14] and abnormal metabolism [8], [10] in HE in previous studies, the question remains how the basal ganglia affect other brain system and is affected by other brain regions in this disease. To address this problem, in this rs-fMRI study, we aimed to evaluate altered directional connectivity patterns from and to the basal ganglia in the low-grade HE by using Granger causality analysis (GCA). GCA origins from the field of economics and has been widely used for time-directed prediction between BOLD-fMRI time series, and revealing the causal effects among brain regions [17], [18], [19]. Taking into account that the globus pallidus are the mainly affected regions within the basal ganglia in HE, we chosen bilateral globus pallidus as seed regions and hypothesized that effective connectivity networks of them were disrupted in low-grade HE patients. To the best of our knowledge, the present study is the first to examine the time-directed dynamic relations in HE with GCA.

## Materials and Methods

### Subjects

This study was approved by the Medical Research Ethics Committee of Jinling Hospital and Clinical School of Medical College at Nanjing University. Thirty five low-grade HE patients (28 male, 7 women, mean age: 53.86±7.82 years) and thirty five age-and gender-matched healthy controls (28 male, 7 women, mean age: 50.40±9.19 years) were included in this study, after giving the written informed consents. All participants: 1) were right-handed, with age 18 years or older. 2) had no reported history of brain injury and any psychiatric disorder; 3) had no reported substance abuse; and 4) had no physical limitations that prohibited them from finishing the MR exam, and no translation more than 1.0 mm or rotation than 1.0° during MR scanning.

The patients were recruited from hospitalized patients in Jingling hospital with criterion as follows: the patients with clinical proven hepatic cirrhosis, without comatose, had abnormal neuropsychological tests scores. The overt HE was graded with the West Haven Criteria [20]. Of these patients, 28 were defined as minimal HE (MHE) and 7 as grade I HE. We combined these two entities into one group according to the recent classification by the International Society for Hepatic Encephalopathy and Nitrogen Metabolism (ISHEN) [21], [22], in which both the MHE and grade 1 HE are named as covert HE for simplifying in practical use. The healthy controls were recruited from the local area by means of poster advertisement. They had no diseases of the liver and other systems, with no abnormal findings in abdominal ultrasound scans and conventional brain MR imaging. All controls underwent neuropsychological tests that also performed on HE patients before the MR scanning. No laboratory tests were performed thus unavailable for them.

### Neuropsychological tests

The definition of HE was based on the West Haven criteria [20]. MHE was diagnosed according to the recommendation by the working party of 11^th^ World Congress of Gastroenterology in Vienna in 1998 [20]. The test battery includes number connecting-A (NCT-A) and digit symbol test (DST), which are recommended by the working party. When the scores of at least one test were beyond 2SD (standard deviation) of mean value of age-matched healthy controls, the cirrhotic patients without previous and current overt HE could be regarding having MHE [2], [23].

### Laboratory Examinations

Laboratory parameters including prothrombin time, protein metabolism tests, venous blood ammonia were obtained from all patients to assess the severity of liver disease, within one week before MR scanning. The grade of hepatic function was determined according to the Child-Pugh score [24], [25]. The score system considered five variables, i.e., ascites, encephalopathy, prothrombin time, and serum levels of bilirubin and albumin, and assigned a score ranging from 1 to 3 to each variable. Of these 35 low-grade HE patients, 12 patients had Child-Pugh grade A, 19 patients had Child-Pugh grade B, and 4 had Child-Pugh grade C.

### MRI data acquisition

MRI data were acquired on a 3 Tesla MR scanner (TIM Trio, Siemens Medical Solutions, Erlangen, Germany). All the patients and healthy controls were instructed to close their eyes but be awake during the resting-state functional MR imaging examination. Foam pad was used to minimize the head motion of all subjects. Axial anatomical images were acquired using a T1-FLASH sequence (TR/TE = 350 ms/2.46 ms, matrix = 320×256, field of view (FOV) = 240×240 mm^2^, slice thickness/gap = 4.0 mm/0.4 mm, 30 slices covered the whole brain). Functional images were then obtained aligned along the anterior commissure-posterior commissure line with a single-shot, gradient-recalled echo planar imaging sequence (TR/TE = 2000 ms/30 ms, FOV = 240×240 mm^2^, flip angle = 90°, matrix = 64×64, voxel size = 3.75×3.75×4 mm^3^). A total of 250 brain volumes were collected, resulting in a total scan time of 500 s.

### Data preprocessing

Data were pre-processed using SPM8 software package (http://www.fil.ion.ucl.ac.uk/spm). The first 10 images were excluded for magnetization to reach equilibrium. The remaining 240 consecutive volumes were used for data analysis. Slice-timing adjustment and realignment for head-motion correction were performed. No translation or rotation parameters in any given data set exceeded 1.0 mm or 1.0°. We also evaluated the group differences in translation and rotation of head motion according to the following formula [26]: 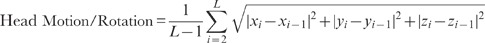 where *L* is the length of the time series (*L* = 240 in this study), *x_i_*, *y_i_* and *z_i_* are translations/rotations at the *i*th time point in the *x*, *y* and *z* directions, respectively. The results showed that the two groups had no significant differences in image quality (two sample *t* test, *t* = 1.489, *P* = 0.141 for translational motion, and *t* = 1.391, *P* = 0.169 for rotational motion).

The functional images were then spatially normalized to standard stereotaxic coordinates of the standard Montreal Neurological Institute (MNI) and resampled into voxel size of 3×3×3 mm^3^, and then smoothed by convolution with an isotropic Gaussian kernel of 8 mm FWHW to decrease spatial noise. To further reduce the effects of confounding factors unlikely to be involved in specific regional correlation, we also removed several sources of spurious variance by linear regression, including six head motion parameters, and average signals from cerebrospinal fluid, white matter according to previous fMRI studies [27], [28]. Then, the residual time series were band filtered (0.01–0.08 Hz) using the Resting State fMRI Data Analysis Toolkit (REST) Version 1.6 [29] (http://www.restfmri.net).

### Definition of seed regions

In individual rs-fMRI data analysis, we used the bilateral globus pallidus as seed regions. These seed regions were selected using the WFU PickAtlas Tool Version 3.0 (http://fmri.wfubmc.edu/software/PickAtlas) [30], which was used to define the reference time series and have been applied in previous rs-fMRI studies [31].

### Effective connectivity analysis

In the current study, we used Granger causality to describe the effective connectivity between the reference time series of the seed regions (left and right globus pallidus, respectively) and the time series of each voxel within the whole brain. Voxel-wise GCA on the residual-based *F* was performed using the REST-GCA [32] in the REST toolbox. GCA is a technique that originally developed to evaluate causal relation between two time series in the field of economics [33]. It is based on the idea that, given two time series *x* and *y*, if knowing the past of *y* is useful for predicting the future of *x* then *y* must have a causal influence on *x*. In this study, the time series of the globus pallidus was defined as the seed time series *x*, and the time course of voxels within the whole brain are defined as *y*. The linear direct influence of *x* on *y* (*F_x_*_→*y*_), and the linear direct influence of *y* on *x* (*F_y_*_→*x*_) were calculated voxel by voxel across the brain. The residual-based *F* was transformed to normal distributed *F*′, then *F*′ of each voxel was standardized to *Z* score (*Z_x_*_→*y*_ and *Z_y_*_→*x*_, subtracting the global mean *F*′ values, then being divided by standard deviation) [32].

### Statistical analysis

#### Group analysis of functional connectivity of globus pallidus

Within each group, mean values of the *Z_x_*_→*y*_ and *Z_y_*_→*x*_ maps were calculated. All eight Granger causality maps were obtained, with four for each direction and four for each group (the left globus pallidus with *Z_x_*_→*y*_ and *Z_y_*_→*x*_ and the right globus pallidus with *Z_x_*_→*y*_ and *Z_y_*_→*x*_ for both the patient and healthy control groups). Then a random effect two-sample *t*-test in a voxel-wise manner was then performed to determine the differences of effective connectivity of globus pallidus between two groups, with age and sex importing as covariates. Significant thresholds were set at a corrected *P*<0.05 [multiple correction using false discovery rate (FDR) criterion].

### Pearson correlation analysis of effective connectivity of globus pallidus

To investigate the association between the clinical index and the effective connectivity of globus pallidus in the patients, the regions showing significantly different (increased or decreased) Granger influences between patient and healthy control groups were extracted as regions of interest (ROIs). Mean Granger causality values within these ROIs were correlated against the venous blood ammonia levels, and the neuropsychological performances (scores of NCT and DST) of all patients using the Pearson correlation analysis. Statistical threshold was set at *P*<0.05, uncorrected.

## Results

### Demographics and clinical data

**Table 1** showed the demographics and clinical data of all the 70 participants in our study. All subjects were right-handed. There were no significant differences in gender, age between the low-grade HE and healthy control groups. However, HE patients had worse neuropsychological performances than healthy controls.

**Table 1:** Demographics and clinical data of low-grade HE patients and healthy controls.

Protocols	HC (n = 35)	low-grade HE (n = 35)	*P* value
Sex (M/F)	28/7	28/7	1.00^a^
Age (±SD), y	50.69±9.20	53.86±7.82	0.13^b^
Venous blood ammonia (in µmol/L)	-	63.37±29.85	
Child-Pugh scale			
A	-	12	
B	-	19	
C	-	4	
NCT-A (second)	45.71±7.75	70.52±21.50	<0.001^b^
DST	43.64±10.58	26.87±9.74	<0.001^b^

Quantitative values are expressed as mean ± SD (standard deviation). M = male; F = female; HC = healthy control; HE = hepatic encephalopathy. NCT-A = number connecting-A; DST = digit symbol test; - = unavailable data.

a The *P* value for gender distribution in the two groups was obtained by chi-square test.

b The *P* value for age and neuropsychological tests difference between the two patients groups was obtained by two sample *t* test.

### Effective connectivity from the left globus pallidus

**Figure 1:**
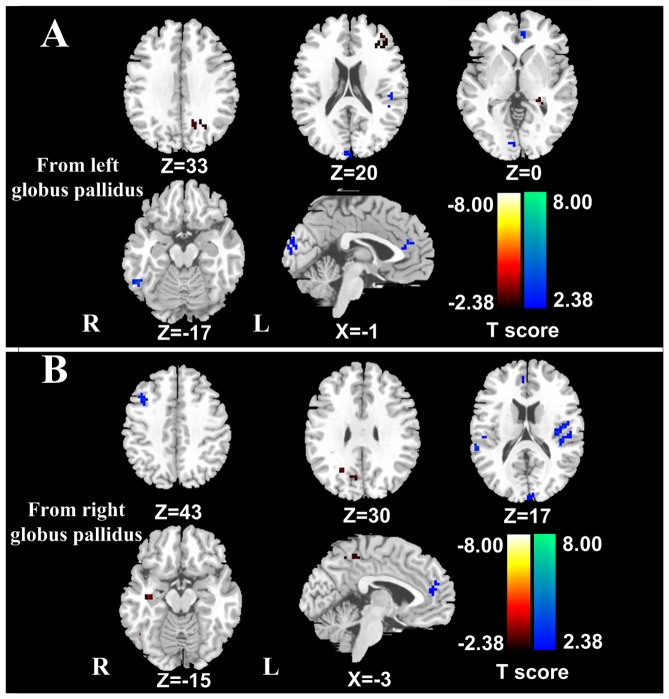
Altered effective connectivity from the globus pallidus to other brain regions in low-grade HE patients (*P*<0.05, FDR corrected). Compared with healthy controls, HE patients show decreased effective connectivity from the left globus pallidus to the left ACC, bilateral cuneus, right ITG, left STG, and increased effective connectivity to the left precuneus, left MFG and bilateral parahippocampal gyri (1A). Patients exhibit decreased effective connectivity from the right globus pallidus to the right ACC, left cuneus, bilateral STG and MFG, and increased effective connectivity to the right precuneus, right MFG, and right parahippocampal gyrus (1B). The hot and cold colors indicate the brain regions that show significantly increased and decreased effective connectivity, respectively. HE = hepatic encephalopathy; FDR = false discovery rate; ACC = anterior cingulate cortex; ITG = inferior temporal gyrus; STG = superior temporal gyrus; MFG = middle frontal gyrus.

Compared with healthy controls, patients with low-grade HE demonstrated significantly decreased effective connectivity from the left globus pallidus to seveal brain regions, including the left anterior cingulate cortex (ACC), bilateral cuneus, right inferior temporal gyrus (ITG), left superior temporal gyrus (STG), and increased effective connectivity from the left globus pallidus to the left precuneus, left middle frontal gyrus (MFG) and bilateral parahippocampal gyri (*P*<0.05, FDR corrected) (**Figure 1A** **,** **Table 2**).

**Table 2:** Altered effective connectivity from the left globus pallidus to the other brain regions in low-grade HE patients.

Regions	Hem	BA	MNI coordinates (mm)	ΔVol (mm^3^)	Maximal *t* value
			(x, y, z)		
**Decreased**		…			
ACC	L	32	−12,45,9	13	−2.56
Cuneus	R	18	12,−87,−15	10	−2.50
Cuneus	L	18	−6,−102,12	16	−2.49
ITG	R	37	57,−57,−21	27	−2.47
STG	L	40	−51,−24,21	11	−2.39
**Increased**					
PHG	L	27	−27,−36,−3	10	+3.06
MFG	L	46	−39,45,18	12	+3.03
PHG	R	35	24,−3,−30	10	+2.95
Precuneus	L	7	−24,−60,33	23	+2.70

Positive sign represents increase, and negative sign represents decrease. HE = hepatic encephalopathy; FDR = false discovery rate; Hem = hemisphere; BA = Brodmann's area; MNI = Montreal Neurological Institute; ΔVol: volume difference; ACC = anterior cingulate cortex; ITG = inferior temporal gyrus; STG = superior temporal gyrus; PHG = parahippocampal gyrus; MFG = middle frontal gyrus.

### Effective connectivity from the right globus pallidus

When compared with the healthy controls, low-grade HE patients exhibited significantly decreased effective connectivity from the right globus pallidus to seveal brain regions that included the right ACC, left cuneus, bilateral STG and MFG, and increased effective connectivity to the right precuneus, right MFG, and right parahippocampal gyrus (*P*<0.05, FDR corrected) (**Figure 1B** **,** **Table 3**).

**Table 3:** Altered effective connectivity from the right globus pallidus to the other brain regions in low-grade HE patients.

Regions	Hem	BA	MNI coordinates (mm)	ΔVol (mm^3^)	Maximal *t* value
			(x, y, z)		
**Decreased**		…			
ACC	R	32/9	3,48,21	12	−2.42
MFG	R	8/6	39,15,48	59	−2.41
STG	L	13/41	−54,−18,15	11	−2.40
STG	R	22/41	51,−24,21	11	−2.39
Cuneus	L	18	−3,−96,6	11	−2.39
**Increased**					
PHG	R	35	36,−15,−15	14	+3.67
Precuneus	R	7/5	6,−57,51	34	+3.18
MFG	L	11	−30,45,−6	12	+3.18

Positive sign represents increase, and negative sign represents decrease. HE = hepatic encephalopathy; FDR = false discovery rate; Hem = hemisphere; BA = Brodmann's area; MNI = Montreal Neurological Institute; ΔVol: volume difference; ACC = anterior cingulate cortex; MFG = middle frontal gyrus; STG = superior temporal gyrus; PHG = parahippocampal gyrus.

### Effective connectivity to the left globus pallidus

**Figure 2:**
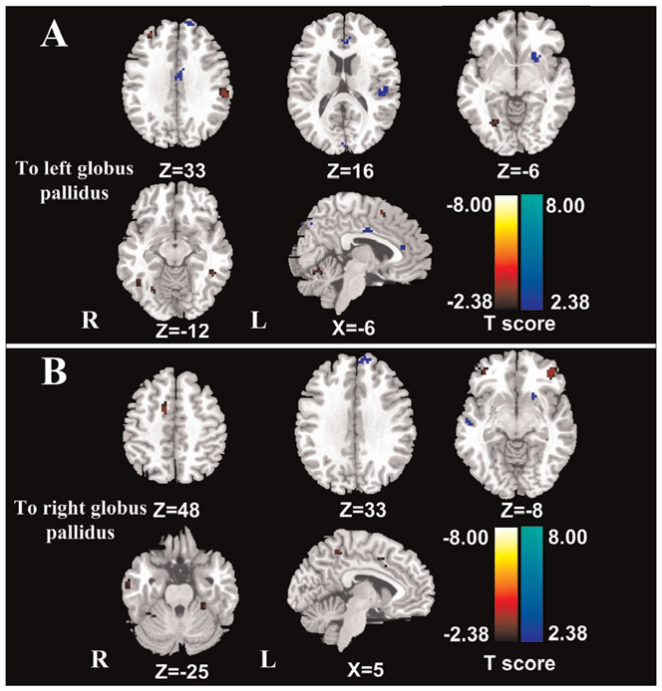
Altered effective connectivity from the other brain regions to the globus pallidus in low-grade HE patients (*P*<0.05, FDR corrected). The left ACC, right cuneus, MCC, left SFG, left STG and putamen show decreased effective connectivity to the left globus pallidus in patients when compared to healthy controls. Moreover, the left SMA, left IPL, right MFG, bilateral ITG and cerebellum display increased effective connectivity to the left globus pallidus (2A). In patients, the left SFG, bilateral STG, right MTG, and left putamen exhibit decreased effective connectivity to the right globus pallidus. Moreover, the right precuneus, right SMA, bilateral MFG, left MTG, right ITG and bilateral cerebellum demonstrate increased effective connectivity to the right globus pallidus (2B). HE = hepatic encephalopathy; FDR = false discovery rate; ACC = anterior cingulate cortex; MCC = middle cingulate cortex; SFG = superior frontal gyrus; STG = superior temporal gyrus; SMA = supplementary motor area; IPL = inferior parietal lobule; MFG = middle frontal gyrus; ITG = inferior temporal gyrus; MTG = middle temporal gyrus.

In patients, several brain regions showed decreased effective connectivity to the left globus pallidus, including the left ACC, right cuneus, middle cingulate cortex, left SFG, left STG and putamen. Moreover, the left supplementary motor area (SMA), left inferior parietal lobule, right MFG, bilateral ITG and cerebellum displayed increased effective connectivity to the left globus pallidus (*P*<0.05, FDR corrected) (**Figure 2A** **,** **Table 4**).

**Table 4:** Altered effective connectivity from the other brain regions to the left globus pallidus in low-grade HE patients.

Regions	Hem	BA	MNI coordinates (mm)	ΔVol (mm^3^)	Maximal *t* value
			(x, y, z)		
**Decreased**		…			
MCC	L	24	−9,0,33	14	−2.59
SFG	L	9	−12,54,21	22	−2.49
ACC	L	24/32	−3,33,15	11	−2.48
STG	L	40/41	−54,−27,15	13	−2.44
Putamen	L	…	−21,18,−6	11	−2.43
Precuneus	L	7	−9,−72,42	31	−2.42
Cuneus	R	18/17	3,−87,12	22	−2.41
**Increased**					
IPL	L	40	−57,−27,36	21	+4.08
SMA	L	6	−6,9,54	15	+3.49
Cerebellum_6	R	…	30,−45,−30	20	+3.46
ITG	R	37	42,−48,−21	10	+3.39
MFG	R	11	30,45,−6	14	+3.18
ITG	L	37	−48,−45,−12	18	+3.20
Cerebellum_6	L	…	−30,−45,−27	12	+3.06

Positive sign represents increase, and negative sign represents decrease. HE = hepatic encephalopathy; FDR = false discovery rate; Hem = hemisphere; BA = Brodmann's area; MNI = Montreal Neurological Institute; ΔVol: volume difference; MCC = middle cingulate cortex; SFG = superior frontal gyrus; ACC = anterior cingulate cortex; STG = superior temporal gyrus; IPL = inferior parietal lobule; SMA = supplementary motor area; ITG = inferior temporal gyrus; MFG = middle frontal gyrus.

### Effective connectivity to the right globus pallidus

In patients, the left SFG, bilateral STG, right MTG, and left putamen showed decreased effective connectivity to the right globus pallidus. Moreover, the right precuneus, right SMA, bilateral MFG, left MTG, right ITG and bilateral cerebullum exhibited increased effective connectivity to the right globus pallidus (*P*<0.05, FDR corrected) (**Figure 2B** **,** **Table 5**).

**Table 5:** Altered effective connectivity from the other brain regions to the right globus pallidus in low-grade HE patients.

Regions	Hem	BA	MNI coordinates (mm)	ΔVol (mm^3^)	Maximal *t* value
			(x, y, z)		
**Decreased**		…			
SFG	L	9	−9,51,33	25	−2.49
MCC	L	24	−3,−9,36	10	−2.47
STG	R	41	42,−27,9	13	−2.41
Putamen	L	…	−18,18,−3	12	−2.40
**Increased**					
MFG	L	10/11	−45,45,9	32	+3.94
SMA	R	24/6	9,0,45	25	+3.75
Cerebellum_4/6	L		−36,−45,−33	11	+3.64
MTG	L	39	−54,−60,12	16	+3.55
ITG	R	20	57,−12,−24	13	+3.38
Cerebellum_6	R		30,−48,−24	10	+3.35
Precuneus	R	7	0,−51,54	15	+3.31
MFG	R	10/11	36,45,−9	29	+3.27

Positive sign represents increase, and negative sign represents decrease. HE = hepatic encephalopathy; FDR = false discovery rate; Hem = hemisphere; BA = Brodmann's area; MNI = Montreal Neurological Institute; ΔVol: volume difference; SFG = superior frontal gyrus; MCC = middle cingulate cortex; STG = superior temporal gyrus; MFG = middle frontal gyrus; SMA = supplementary motor area; MTG = middle temporal gyrus; ITG = inferior temporal gyrus.

### Correlations results

**Figure 3:**
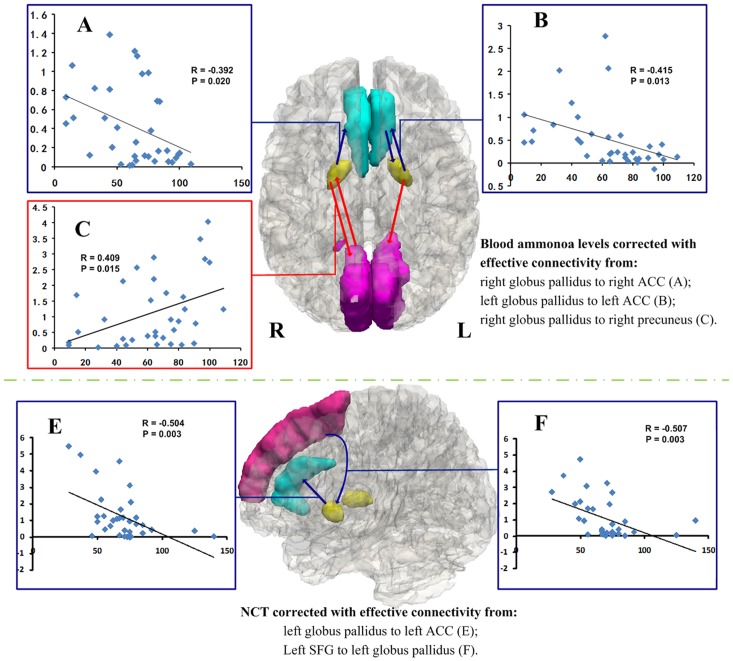
Correlations results between the altered effective connectivity of the globus pallidus and blood venous ammonia and neuropsychological performances in low-grade HE patients (*P*<0.05, uncorrected). Pearson correlation analyses reveals that the blood venous ammonia levels of low-grade HE patients negatively correlate with the decreased effective connectivity from the bilateral globus pallidus to the iso-lateral ACC (3A/B), and positively correlate with the influence from the right globus pallidus to the right precuneus (3C). The other regions with aberrant effective connectivity show no correlation with venous blood ammonia levels. In addition, the number connectivity test scores in patients negatively correlated with the effective connectivity from the left globus pallidus to left ACC, and from left superior frontal gyrus to left globus pallidus. HE = hepatic encephalopathy; ACC = anterior cingulate cortex.

Pearson correlation analyses revealed that the blood venous ammonia levels of low-grade HE patients negatively correlated with the decreased effective connectivity from the bilateral globus pallidus to the iso-lateral ACC (left side: *R* = −0.415, *P* = 0.013; right side: *R* = −0.392, *P* = 0.020), and positively correlated with the influence from the right globus pallidus to the right precuneus (*R* = 0.409, *P* = 0.015) (**Figure 3**). In addition, the number connectivity test scores in patients negatively correlated with the effective connectivity from the left globus pallidus to left ACC (*R* = −0.504, *P* = 0.003), and from left superior frontal gyrus to left globus pallidus (*R* = −0.507, *P* = 0.005). The other regions with aberrant effective connectivity showed no significant correlation with blood ammonia and neuropsychological performances.

## Discussion

The present resting-state fMRI study with Granger causality analysis demonstrated that low-grade HE patients had abnormal directionality of influence both from and to the globus pallidus; moreover, the disturbed connectivity between the globus pallidus and the ACC, as well as the precuneus was associated with the clinical index of HE (blood ammonia, neuropsychological performance). To the best of our knowledge, this is the first study to examine the causality interactions of basal ganglia in HE patients by using fMRI.

### Effective connectivity revealed by Granger causality analysis

GCA used in the present study is a method based on multiple linear regression for investigating whether the past value of one time series could correctly predict the current value of another [18]. In the past few years, GCA has been applied on neuroimaging studies to reveal the causal effects among brain regions, first on electroencephalography (EEG) and magnetoencephalography (MEG) data [18], [34], [35], and later on fMRI data [17], [27], [36]. Moreover, there have been only a few reports of GCA in patients including social anxiety disorder [27], Alzheimer's disease [37], and major depressive disorder [36]. Convergent neuroimaging evidence has indicated that many brain regions show abnormal metabolism or activity in HE patients, suggesting there is an alteration of the cortico-striato-thalamic pathway in the HE patients [7], [8]. In addition, a very recent fMRI study demonstrated disrupted functional connectivity between the basic ganglia and many other brain regions in HE patients [14]. However, the causality interactions (effective connectivity) between the basic ganglia and other affected brain regions could not be inferred from that study. This resting-state fMRI study with GCA extends our understanding of the influences of the basic ganglia in the neuropathophysiology of HE.

### Disrupted effective connectivity from and to the globus pallidus

This study revealed mutually decreased influence between the globus pallidus and the ACC, cuneus, which was partically supported by the negative correlation between the influence from the globus pallidus to the ACC. ACC is regarded as a core region to be involved in attention which severs to regular both cognitive and emotional processing [38]. Interestingly, attention defect is a fundamental aspect of HE [3], [4], and abnormal ACC function in cirrhotic patients with or without HE had been widespreadly reported by studies using PET [39], fMRI [40], and MRS [41]. In particular, a recent resting-state fMRI exhibited a decreased functional connectivity between the basal ganglia and the ACC [14]. That study focused on the temporal synchrony or correlation between the basal ganlia and the ACC, our study emphasized the effective influence between them. In addition, one recent study [42] showed that the transjugular intrahepatic portosystemic shunt (TIPS) had different short-term and long-term effects on cirrhotic patients' ACC activity, and the changes of ACC activity might have a potential role in predicting the development of postsurgical HE. Taken together, these findings of abnormal ACC function may account for the attentional and cognitive deregulation in HE patients.

Cuneus is regarded as a core region to visual and spatial attention, and inhibitory control [43]. Zafiris et al. reported an impaired neural interaction in early HE patients between the cuneus and many other brain regions in a task-driven fMRI study by using critical flicker frequency (CFF) test [44]. Ni et al. found that the cuneus exhibited decreased brain regional homogeneity (ReHo) in MHE patients, and the decreased ReHo values were associated with their neuropsychological performances [45]. A recent rs-fMRI study by Zhang et al. also showed a decreased functional connectivity between the pallidum and the cuneus [14]. So our finding of decreased influence between the globus pallidus and the cuneus is complementary to previous studies, the findings in all these studies indicate that the disturbance of basal ganglia-cuneus may partially mediate the attention and control dysfunction in HE patients [14].

We also found mutually increased influence between the globus pallidus and the precuneus. Precuneus is thought to be engaged in visuo-spatial imagery, episodic memory retrieval and self-processing operations [46]. Chen et al. reported an increased regional activity in precunecus in MHE patients in a rs-fMRI study with regional homogeneity measurement [47]. In addition, Qi et al. recently demonstrated an increased functional conectivity of the precunecus with other brain regions within the brian default mode network in a fMRI study in 14 MHE patients [40], and speculated it as a compensatory phenomenon in the early stage of HE. The increased influence from pallidum to precunecus in this study may also be interpreted as a compensatory mechanism, which needs to be confirmed in further studies. Our finding of positive correlation between the effective connection from pallidum to the precunecus and blood ammonia levels also supported this compensatory hypothesis.

In this study, we also found decreased influence from the globus pallidus to the right inferior temporal gyrus (ITG), and left superior temporal gyrus (STG). The ITG is involved in visual perception [48], and the STG subserves language processes [49]. A decreased interaction between globus pallidus and these regions may account for the impaired visual function which is often reported in the early stage of HE [4], [44]. Furthermore, increased influences from pallidum to bilateral middle frontal gyri and parahippocampal gyri were found in our study. Parahippocampal gyrus is a part of the limbic system, for which one previous PET study has showed an increased regional cerebral blood flow (rCBF) in early HE patients [50]. This perfusion increase in the limbic circuit was interepted as a compensatory response to premptor, motor, and complex attentional deficts in patients [50]. Validity of our findings and whether the increased influence indicated a compensatory mechanism need to be confirmed in further studies.

As for the influence from other brain regions to the pallidum in the present study, many frontal and temporal gyri, and cerebellum were found to show either decreased or increased influence to the globus pallidus, similar to the findings in prior neuroimaging studies of HE [9], [51]. We speculated that in the early phase of HE, there co-exist both impairment and compensatory mechanisms, which was also suggested in a previous fMRI study [40]. Our findings added important insights into understanding the brain functional impairment in early HE patients.

Our study has some limitations. First, the meaning of the GCA in the resting-state fMRI is not fully understood, and there are some shortages of GCA, e.g., during the process of GCA, spurious influences might be obtained because of the variability in the shape and latency of hemodynamic response functions (HRFs) in different brain regions and different subjects [52], and the slow dynamics of the BOLD signal (2 s used here) may induce some missing of rapid causal influences [53]. However, many scholars believe it reflects time-directed influence between a seed region and the rest of the brain [18], [36], [53]. To elucidate the association between effective connectivity and neuronal activity, further study combing fMRI and electrophysiology is needed to perform. Secondly, potential effects of medication such as diuretics for controlling ascites in some patients, might have an affect on the statistical analysis and results of this study. Further studies in a larger population with better treatment controls are needed to verify these findings. In addition, we only select the globus pallidus as seed region within the basal ganglia, the results could not extended to other basal ganglia regions, e.g., putamen, caudate, and substantial nigra. Thirdly, the study include only low-grade HE patients, the classification from low-grade to high-grade HE should be clarified in further studies. Finally, in this preliminary study investigating the influence between regions in HE patients, a lenient significnat threshold was used in the correlation analysis between effectivity and clinical index. This correlation analysis was exploratory in nature, and a stricter threshold should be used in the further studis.

## Conclusions

In conclusion, we detected disrupted effective connectivity network of basal ganglia in low-grade HE patients, with abnormal directionality of influence both from and to the globus pallidus. Our findings may help to understand the neurophysiological mechanisms underlying the HE.
